# Effects of Upregulation of Hsp27 Expression on Oocyte Development and Maturation Derived from Polycystic Ovary Syndrome

**DOI:** 10.1371/journal.pone.0083402

**Published:** 2013-12-31

**Authors:** Lingbo Cai, Xiang Ma, Shan Liu, Jinjuan Liu, Wei Wang, Yugui Cui, Wei Ding, Yundong Mao, Huiping Chen, Jie Huang, Zuomin Zhou, Jiayin Liu

**Affiliations:** State Key Laboratory of Reproductive Medicine, Clinical Center of Reproductive Medicine, First Affiliated Hospital, Nanjing Medical University, Nanjing, China; Institute of Zoology, Chinese Academy of Sciences, China

## Abstract

Heat shock protein 27 (Hsp27) is a heat shock protein family member which can inhibit apoptosis. Our previous studies reported down-regulated Hsp27 in ovarian tissue derived from women with polycystic ovary syndrome (PCOS) however, the exact effect of Hsp27 on oocyte maturation and developmental competence in PCOS is unclear. The effect of Hsp27 over-expression was studied in vitro using oocytes derived from PCOS patients. An artificial GFP-plasmid was injected into human oocyte to increase Hsp27 protein level. Oocyte maturation was evaluated by morphological observation. Mature oocytes were fertilized by intracytoplasmic sperm injection (ICSI) and embryonic developmental competence was evaluated. Critical apoptotic factors and cytokines were measured at both the mRNA and protein level. Our results revealed that Overexpression of HSP27 lowered the maturation rate of oocytes derived from PCOS patients. Meanwhile, fertilization rate and high quality embryo rate were similar between the Hsp27 overexpressing group and controls; however, the blastocyst formation rate in this group was significantly higher than control. Expression analysis revealed that the oocyte-secreted factors, *BMP15* and *GDF9*, and the apoptotic-related regulators, Caspase 3, 8 and 9, were all significantly decreased in Hsp27 overexpressing oocytes. In conclusion, upregulation of Hsp27 inhibits oocyte maturation from PCOS patients, but improves embryonic developmental potential.

## Introduction

Polycystic ovary syndrome (PCOS) is the most common cause of female infertility, affecting 5 to 10% of women during their reproductive age [1]. It is characterized by ovarian hyperandrogenism, insulin resistance and dysregulation of paracine factors, all of which can perturb the intrafollicular environment [2]–[4]. The ovary of individuals with PCOS experiences abnormal apoptotic activity and folliculogenesis, making it a clinical pathological model for studying oocyte maturation and development. Currently, the cause and pathophysiological mechanism of PCOS is unclear; however, evidence indicates there is an imbalance between pro-apoptotic and anti-apoptotic factors within the ovary [5].

Heat shock protein 27 (Hsp27), a member of the small heat shock protein family, is an apoptotic regulator which can inhibit apoptosis [6]. As a molecular chaperone protein, Hsp27 is involved in cellular protection in response to a variety of stresses, such as heat shock, toxicants, injury, and oxidative stress [7]. Emerging evidence indicates that Hsp27 has strong anti-apoptotic properties, mediated by a direct interaction with the caspase activation components in apoptotic pathways, consequently exerting protective effects in apoptosis-related injuries [8], [9]. For example, Hsp27 has been shown to protect cells against apoptosis by binding with cytochrome C, inhibiting the activation of Caspase 9 and blocking the extrinsic Fas- and TNF-mediated apoptotic pathways [10]–[13]. Interestingly, we previously found that Hsp27 was mainly expressed in human oocytes, and was downregulated in ovarian tissue isolated from women with PCOS [14]. We also found that downregulation of Hsp27 improves oocyte maturation in mice, while increasing early stage apoptosis in oocytes by inducing the activation of the extrinsic, caspase 8-mediated, apoptotic pathway [15]. Despite these important findings, the exact effect of Hsp27 on oocyte maturation and developmental competence in PCOS has not been clarified.

We hypothesize that downregulation of Hsp27 expression might simultaneously affect multiple signaling pathways in the ovary, contributing to the abnormal oocyte development characteristic of PCOS. In our previous studies, we established the differential proteomic profile of PCOS ovarian tissue by two-dimensional gel electrophoresis and mass spectroscopy [14]. Hsp27 was one of the differentially-expressed proteins identified in this study which is primarily expressed in oocytes; we interpreted this data to suggest that Hsp27 may participate in the molecular pathogenesis of PCOS. Based on the importance of oocytes in follicular development and the low expression of Hsp27 in PCOS oocytes, we hypothesized that Hsp27 could affect oocyte development, maturation, and developmental competence. Our previous study showed that HSP27 levels affect maturation and expression of apoptotic factors in mouse oocytes in vitro [15]. In the present study, the effect of overexpression of Hsp27 was investigated in vitro, using oocytes isolated from patients with PCOS. to investigating the effect of Hsp27 on oocyte development and maturation, as well as its effect on fertilization and embryonic developmental competence in vitro. The results of this study provide an improved insight into developmental disorders of the oocyte, particularly with regard to the pathophysiology of PCOS.

## Materials and Methods

In our study, all participants provide their written informed consent to participate in this study. And our study was approved by ethics committee, First Affiliated Hospital, Nanjing Medical University.

### Isolation of immature oocytes from patients with PCOS

Immature oocytes were donated by patients with PCOS from The Center of Clinical Reproductive Medicine, First Affiliated Hospital of Nanjing Medical University. These patients failed in ovulation induction at least 6 times and were untreated with any medicines before receiving ovarian drilling. A total of 29 patients donated their immature oocytes, and all patients signed informed consents forms. The mean patient age was 27.8±2.4 years, duration of infertility was 3.8±2.3 years, and mean BMI was 25.1±2.1. Cumulus-oocyte complexes were retrieved from ovaries by small follicle (6 to 8 mm) aspiration under ultrasound guidance. The programme was approved by the hospital's ethical committee and the local and central governments. Institutional Review Board approval was granted.

### Hsp27 overexpression vector construction

In order to clone HSP27 we designed two set of primers,containing two restriction sites to be used: sense: 5′-GCA GAT CTT ATG ACC GAG CGC CGC GTC C-3′, antisense: 5′-ATG TCG ACT TAC TTG GCG GCA GTC TCA TCG G-3′. The open reading frame of the target gene was amplified by PCR using high-fidelity Taq enzyme (Takara Shuzo Co. Ltd. Kyoto, Japan). The PCR conditions were as follows: 95°C for 5 min, 35 cycles at 95°C for 30 s, 50°C for 30 s, and 72°C 3 for 90 s, followed by a 7 min extension at 72°C. The PCR products were then separated using a 1.5% agarose gel and subcloned into the adenoviral shuttle vector pAdTrack-CMV (Stratagene, Agilent Technologies, Inc.) The recombinant plasmid AdCMV-Hsp27 was verified by PCR, restriction endonuclease digest and gene sequencing. The recombinant adenovirus shuttle plasmid pAdTrack-CMV-hHsp27 was first digested by *Sal*I and *Bgl*II enzyme (Takara Shuzo Co. Ltd. Kyoto, Japan), then measured in 0.8% agar gel electrophoresis. Also we compared our pAdTrack-CMV-hHsp27 gene sequencing results with those available in the GenBank.

### Hsp27 overexpression in human oocytes

The above described vector was used to drive Hsp27 expression in oocytes isolated from patients with PCOS. GV oocytes from PCOS patients were randomly divided into three groups, including the experimental group and two control groups. The experimental group was injected with pAdTrack-CMV-Hsp27; one control group was injected the empty vector (pAdTrack-CMV) and the second control group was untreated. The recombinant plasmid pAdTrack-CMV-Hsp27 or pAdTrack-CMV was microinjected into the nucleus of human GV oocytes from PCOS patients. The injection volume was 5 to 7 pl per oocyte. After injection, all GV oocytes were transferred to IVM medium (SAGE, CooperSurgical Company, Trumbull, USA), at 37°C in 5% CO_2_, 5% O_2_, and 90% N_2_ for culture and maturation. Control GV oocytes without injection were also cultured in IVM medium. Oocyte maturation rate was evaluated by morphological appearance. Oocytes without GVs were scored as Germinal Vesicle Breakdown (GVBD) stage oocytes (stage MI). Progression to the metaphase II (MII) stage was identified by extrusion of the first polar body into the perivitelline space. We used immunofluorescence as a semi-quantitative method to detect the expression of proteins and observe intuitively subcellular location, then evaluated GFP 24 h after injection.

### Determination of mRNA expression by RT-PCR

RT-PCR was used to determine mRNA expression levels in all experiments. RNA was isolated from tissues using an RNeasy micro kit (Qiagen, Valencia, CA). Reverse transcription was carried out using a sensiscript reverse transcription kit (Qiagen, Valencia, CA), with oligo-dT primers at 37°C for 60 minutes. Real-time RT-PCR was performed as previously described [15], all reactions were performed using QuantiTect SYBR green PCR kits (Takara Shuzo Co. Ltd, Kyoto, Japan), using an ABI 7300 cycler (Applied Biosystems, Foster City, CA). Relative quantification of target gene expression was calculated using the 2^−ΔΔCt^ method. In these studies, β-actin and GAPDH were used as reference genes. Primer design was carried out using Primer 5.0. Primer sequences for *BMP15, GDF9, CytC, Caspase 3, 8 and 9* are presented in Table 1.

**Table 1:** Primer sequences used for quantitative real-time PCR reactions.

Gene	GenBank Accession No.	Sequence	Location	Product Size (bp)
Caspase 3	NM_032991	F: 5′-TTCAGAGGGGATCGTTGTAGA -3′	427-4475	184
		R: 5′-AATAACCAGGTGCTGTGGAGTA -3′	89-610	
Caspase 8	NM_001080125	F: 5′-AGGAAAGTTGGACATCCTGAAAA-3′	840-862	174
		R: 5′-TAATGACAATCTCGGACTCTCC-3′	992-1013	
Caspase 9	NM_001229	F: 5′- GCGAACTAACAGGCAAGCA-3′	267-285	144
		R: 5′-CCAAATCCTCCAGAACCAAT -3′	391-410	
Cyt *c*	NM_018947	F: 5′-GGAGGCAAGCATAAGACTGG -3′	215-234	203
		R: 5′-GTCTGCCCTTTCTTCCTTCT -3′	408-427	
Bmp15	NM_005448	F: 5′-CTTCCCTGATGTCTAACGCTTGG- 3′	521-543	217
		R: 5′-GCCTTCCGAATGCTTTTATGAGTA- 3′	714-737	
Gdf 9	NM_005260	F: 5′- CAAGGGCAGTTGGACATCGGTA -3′	1160-1181	139
		R: 5′-TCAATGGTCAAAACACTCAAGG- 3′	1277-1298	
GAPDH	NM_002046	F:5′-GAAGGTCGGAGTCAACGGATTT-3′	114-135	223
		R: 5′-CTGGAAGATGGTGATGGGATTTC-3′	314-336	

### Assessment of fertilization rate and embryonic developmental potential

The effect of experimental overexpression of Hsp27 in oocytes from PCOS patients on fertilization rate and embryonic developmental potential was assessed. After oocyte maturation, the MII phase oocytes in each group were fertilized by intracytoplasmic sperm injection (ICSI) and grown in embryo culture medium. Sperm was donated by patients who were undergoing conventional in vitro fertilization therapy. They given informed consent before donated. Fertilization rate was observed after 16 to 18 h after ICSI, with the presence of two pronuclei and two polar bodies signifying normal fertilization.

Zygotes were cultured in embryo Cleavage culture medium (SAGE, CooperSurgical Company, Trumbull, USA), and embryonic development was observed every 24 h. The quality of the embryos was categorized according to developmental stage and the presence of anucleate fragments, as described by Van den Abbeel and colleagues and Karlstrom [16], [17]. In brief: grade 1, even-sized blastomeres without any fragmentation; grade 2, blastomeres of equal or unequal size and minor (<20%) cytoplasmic fragments; grade 3, blastomeres of equal and unequal size and medium cytoplasmic fragments (20 to 50%); grade 4, fragments or blastomeres of unequal size and major cytoplasmic fragments (>50% of blastomere volume). Grade 1 and 2 were considered to by high quality embryos. To assess the developmental potential of oocytes, embryos were moved to blastocyst culture medium (SAGE, CooperSurgical Company, Trumbull, USA) for further culture for 2/3 days and blastocyst formation was observed at day 5/6. Blastocyst quality on day 5/6 was assessed according to the criteria of Gardner and Schoolcraft [18].

### Statistical analysis

All data are presented as the mean ± standard deviation. A one-way analysis of variance and a log linear model were used to compare the mRNA levels. Chi-square analysis was used to compare the rates of oocyte maturation, fertilization and embryo development. A p-value <0.05 was considered statistically significant.

## Results

### Construction and verification of human Hsp27 expression vector

**Figure 1:**
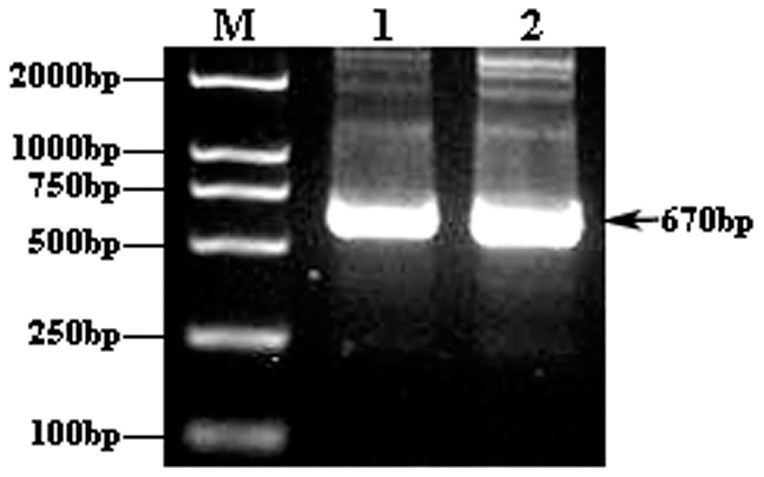
AGE results of PCR amplified products of human HSP27 gene. M: Molecular Weight Marker of DL2000DNA; 1, 2: PCR amplified products (670 bp) with template concentration of 10×, 100× respectively.

**Figure 2:**
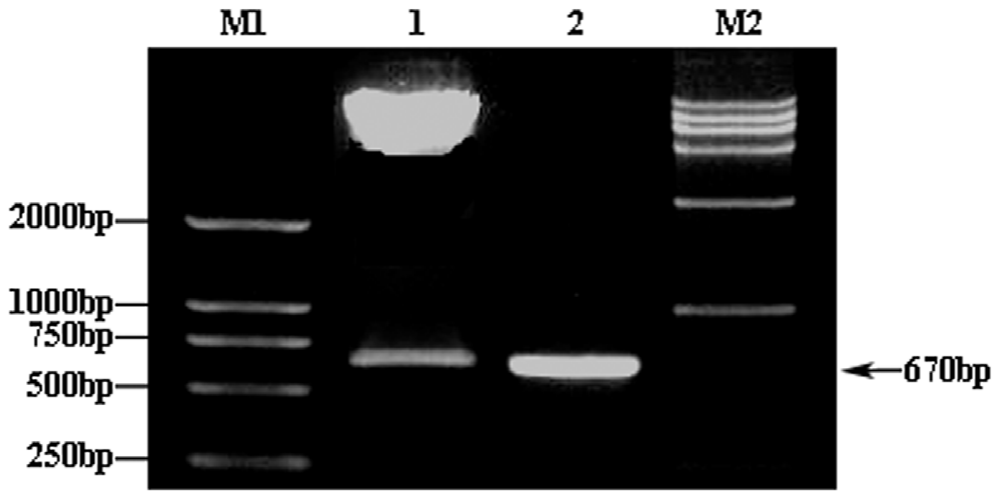
Double-enzyme digestion of pAdTrack-CMV-hHSP27. M1: DL2000 Marker; 1: *Sal*I and *Bgl*II enzyme digestion results of pAdTrack-CMV-hHSP27; M2: *Hind*III enzyme digestionλ-DNA Marker; 2: PCR products of full-length human HSP27.

**Figure 3:**
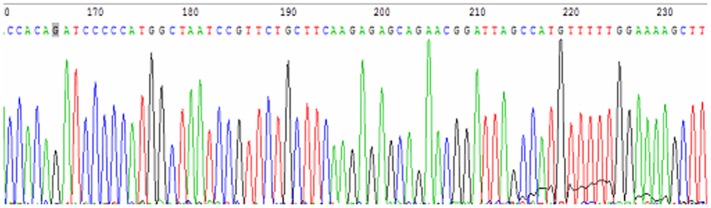
Sequencing results of pAdTrack-CMV-HSP27 (partial).

Analysis of the pADTrack-CMV-hHSP27 vector by agarose gel electrophoresis confirmed the presence of the target gene fragment, with a band visible at the expected size of 670 bp (Figure 1). Following *Sal*I and *Bgl*II restriction enzyme digestion of the recombinant adenovirus plasmid, expected bands were observed at 9.2 kb and 670 bp (Figure 2), demonstrating the successful construction of the pADTrack-CMV-hHSP27 vector. Sequencing of the Hsp27 gene isolated from the vector revealed an identical sequence with that available in the GenBank database (Figure 3).

### Hsp27 overexpression inhibits maturation of human oocytes

**Figure 4:**
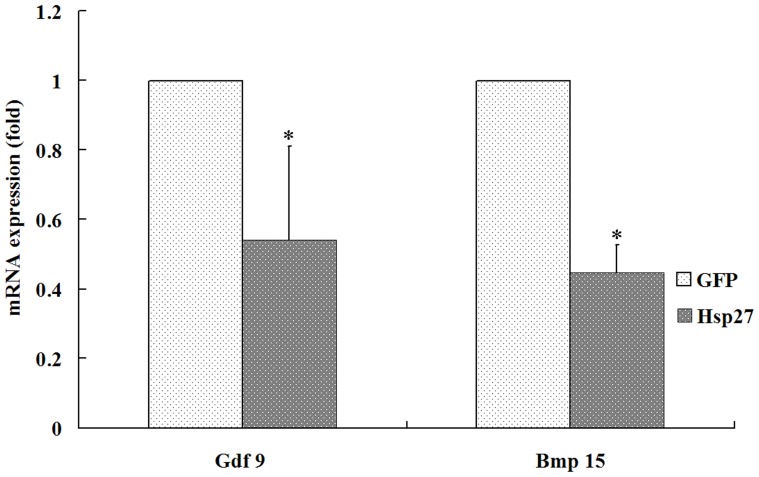
Expression of Bmp15 and Gdf 9 in oocytes after HSP27 up-regulated by Real time RT-PCR. *: P<0.05 *vs.* GFP.

The maturation rate of PCOS oocytes overexpressing Hsp27 and the two control groups are shown in Table 2. Oocytes with successful incorporation of the pADTrack-CMV-Hsp27 vector were identified by the expression of green fluorescent protein, which was included in the vector. The data demonstrate that the maturation rate of pAdTrack-CMV-hHsp27-injected oocytes was significantly lower than those of control oocytes (33.5% versus 55.9% and 61.4%; *P*<0.05). and there was no different between pAdTrack-CMV-Hhsp27 and pAdTrack-CMV-GFP group. The relative expression levels of oocyte-specific factors were assessed by RT-PCR. The mRNA expression level of *BMP15* and *GDF9* was decreased in oocytes overexpressing Hsp27 (Figure 4), which was in accordance with our morphological observations and suggested a close relationship between Hsp27 expression level and oocyte maturation.

**Table 2:** Maturation rate of PCOS oocytes after microinjection of PAdTrack-CMV-hHSP27 at 24 h and 48 h of *in vitro* culture.

		No. of oocytes (%) 24 h			No. of oocytes (%) 48 h		
Treatment	Total	GV	MI	MII	GV	MI	MII
Control	114	21	32	44 (38.6%)	18	14	70 (61.4%)
AdCMV-GFP	213	45	64	79 (37.1%)	29	35	119 (55.9%)
AdCMV-Hsp27	233	86	58	56 (24.9%)*	73	61	78 (33.5%) *

:P<0.05 *vs.* Control.

The fertilization rate, high quality embryo rate and blastocyst formation rate among the three experimental groups was assessed (Table 3). The fertilization rate and the high quality embryo rate at day 3 were not significantly different in Hsp27 overexpressing oocytes, versus control. Blastocyst formation rate in Hsp27 overexpressing oocytes was significant higher than control (41.30% versus 23.53%).

**Table 3:** Maturation, fertilization and embryo development competence of PCOS oocytes after microinjection of PAdTrack-CMV-hHSP27.

Treatment	Total	No. of Metaphase II (MII) oocyte after maturation culture (%)	No. of 2PN zygote	No. of high Grade embryo on day3 (%)	No. of expand blastocyst on day5 and day6 (%)
Control	74	45(60.81)	34(75.56)	20(58.82)	8(23.53)
AdCMV-GFP	141	82(58.16)	62(75.51)	35(56.45)	13(20.97)
AdCMV-Hsp27	151	55(36.42)*	46(83.63)	34(68.89)	19(41.30) *

P<0.05 *vs.* Control.

### Hsp27 overexpression inhibits the expression of apoptotic-related regulators

**Figure 5:**
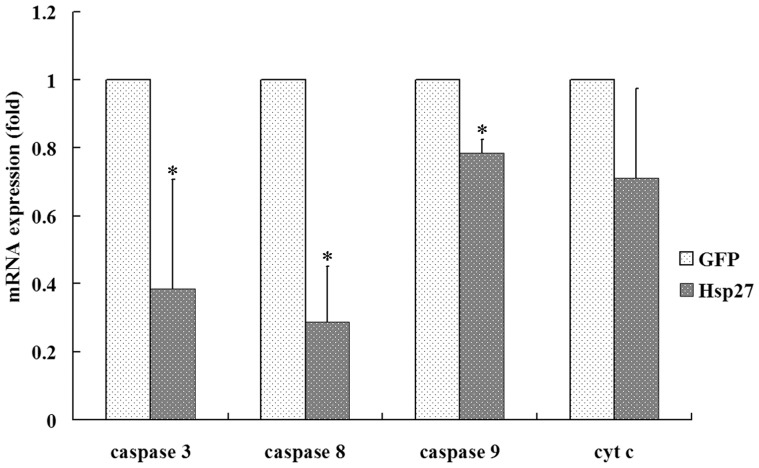
mRNA abundance of apoptosis regulators after HSP27 up-regulated in human PCOS oocytes by Real time RT-PCR. *: P<0.05 *vs.* control.

The mRNA expression levels of Caspase 8, 9 and 3 in oocytes of the pAdTrack-CMV-hHsp27-injected group were significantly lower than that of control (*P*<0.05), while the expression of *CytC* didn't change (Figure 5). We interpreted this data to suggest that Hsp27 overexpression may inhibits the Caspase 8-mediated apoptotic pathway, as well as the intrinsic Caspase 9-mediated pathway.

## Discussion

Patients with PCOS undergoing ovarian stimulation for in vitro fertilization have the increased risk of impaired oocyte developmental competence, implantation failure and pregnancy loss [19]. Moreover, obese PCOS patients experience low oocyte fertilization and failure of embryos to implant in their own uterus or those of their surrogates, implying impaired developmental competence of some PCOS oocytes [20]. It was the goal of this study to better understand the mechanisms underlying altered fertility in patients with PCOS.

Our results showed that Hsp27 upregulation in human PCOS oocytes decreased the rate of oocyte maturation and also decreased the expression of aptoptic related factors. which is consistent with our previous study [15]. Interestingly, Results of the present study also showed that overexpression of Hsp27 didn't affect the rate of fertilization, and even enhanced the embryonic developmental potential of PCOS oocytes.

Hsp27 has been shown to play an important role in a variety of physiological processes, including protein chaperoning, steroidogenesis and protection against apoptosis [6]. Mechanistically, it is known that Hsp27 inhibits apoptosis by sequestering cytochrome c, directly associating with caspase-3, and inhibiting the Fas-induced apoptotic pathway [12], [13]. However, there are few reports about the role of Hsp27 in oocytes, especially human oocytes. Our previous work demonstrated that upregulation of apoptotic-related factors Caspase 8 and Caspase 3 could increase the early apoptotic rate of oocytes, and promoted oocyte maturation in mouse oocytes. Contrastingly, the overexpression of Hsp27 in oocytes was shown to downregulate Caspase 8 and Caspase 3 expression, which depressed the early stages of apoptosis [15]. These findings were further supported by our observation that upregulated Hsp27 expression had an inhibitory effect on oocyte maturation in PCOS patients. Furthermore, it was confirmed in the present study that the level of *BMP15* and *GDF9* decreased when Hsp27 expression was upregulated.

It has been documented that programmed cell death is the mechanism underlying the depletion of oocytes from the ovarian pool [21], [22]. Furthermore, the capacity of an oocyte to undergo apoptosis exists primarily at the early stages of follicular growth (i.e. primordial, primary, and preantral stages) [23]. Apoptosis is recognized as the mechanism of germ cell death and follicle atresia at all stages of ovary development and in each part of the cycle of folliculogenesis. Previously, the imbalance between apoptosis and proliferation has been highlighted as a mechanism which affects the stages of follicle development in PCOS ovaries that may contribute to the polycystic appearance of the ovary [5]. Several studies have found that the early stage of apoptosis is closely related with oocyte development. Li et al. observed that early apoptosis could improve bovine oocyte developmental potential [24]. However, studies on the incidence of apoptosis have produced contradictory results. Matween et al. observed apoptosis occurring before maturation in bovine oocytes [25], while Yuan et al. did not [26]. Apoptosis can also affect oocytes during antral follicle atresia, with the possibility that immature oocytes could be at the early stage of apoptosis before DNA breakdown.

Whether early apoptosis has an effect on developmental capacity of oocytes is unclear. In some animal studies, oocytes in COCs with early signs of atresia (such as slight cumulus expansion and cytoplasmic granulation) exhibited better developmental potential [27]–[29]. Similarly, Hendriksen et al. reported that oocytes from follicles showing signs of atresia underwent similar maturing processes [30]. As we know, apoptosis is a sequential, but reversible process of cell death, with ultimate commitment being made before cells enter into the latter stages of regulation [31]. It has been indicated that the occurrence of early apoptosis in oocytes does not mean that they must develop into late apoptosis, which decreases their developmental competence [32]. In our study, the rate of GV oocyte maturation was decreased and the expression of apoptosis related factors level was lower when Hsp27 was overexpressed, which was consistent with our previous work in maturing mouse oocytes [15]. Hsp27 deficiency has been shown to contribute to an increase in the early apoptotic rate of oocytes. On this basis, we hypothesized that this process may function to block oocyte apoptosis and atresia in vivo, leading to the accumulation of multiple small antral follicles. The data from the present study confirms that the rate of oocyte maturation in PCOS were decreased when the level of Hsp27 expression was upregulated. To confirm this results, mRNA levels of GDF9 and BMP19, growth factors specifically secreted by the oocyte, were analyzed in oocytes overexpressing Hsp27 and were found to be decreased. Furthermore, we investigated the mRNA expression levels of apoptotic-related factors Caspase 3, 8, and 9, and *CytC* to study whether the apoptotic pathways were affected by increased levels of Hsp27 in PCOS oocytes. These results demonstrated that the expression level of Caspase 3 and Caspase 8 were decreased when Hsp27 expression was upregulated.

Oocyte developmental competence following Hsp27 overexpression was also examined in PCOS oocytes. We found that the rate of fertilization and embryonic development was unaffected by Hsp27 overexpression in oocytes isolated from patients with PCOS; however, the blastocyst formation rate in Hsp27 overexpressing oocytes were higher when compared with controls. Due to the difficulty in obtaining oocytes from PCOS patients, our results had a relatively small size sample. As such, further studies are required to elucidate the mechanism underlying Hsp27's effect on oocyte maturation and developmental potential.

To date, the impact of HSP27 on oocytes and embryo development remains unclear. While Polycystic ovary syndrome is associated with decreased antioxidant concentrations, and is thus considered an oxidative state [33]. That could impair oocyte development potential [34]. Hsp27 has the potential to suppress the activation IKK— -βof JNK triggered by diacylglycerol and cytokines thereby protecting IRS-1 from inhibitory serine phosphorylation and helping to preserve insulin sensitivity [35], [36]. Indeed, a role for heat shock in the promotion of insulin sensitivity has been suggested by Hooper. Induction of heat shock proteins may confer broader health benefits to patients who are insulin resistant or diabetic [37]. We Speculate in our study, hsp27 may have also improve oocyte antioxidant condition, to enhance oocyte quality.

In summary, we have demonstrated that Hsp27, an anti-apoptotic factor, may participate in the apoptotic imbalance observed in PCOS oocytes, causing disordered ovulation and activating the extrinsic, capsase 8-mediated apoptotic pathway. Our results show that Hsp27 is involved with the early stage of apoptosis to maintain normal development and maturation of oocytes. In addition, Hsp27 promoted the blastocyst formation rate in patients with PCOS. Further studies will be required to investigate the particular regulatory mechanism of Hsp27 on oocyte maturation and embryo development.
